# Artificial Intelligence-Driven Dairy Quality Assessment: From Advanced Sensing Technologies to Explainable Intelligence

**DOI:** 10.3390/foods15162925

**Published:** 2026-08-20

**Authors:** Xiaodong Song, Zhiran Liang, Congyang Cheng, Rina Wu, Guanjun Dong, Xiaohui Cui, Haohan Ding

**Affiliations:** 1 State Key Laboratory of Dairy Quality Digital Intelligence Monitoring Technology, State Administration for Market Regulation, Hohhot 011517, China; 2 Science Center for Future Foods, Jiangnan University, Wuxi 214122, China; 3 School of Artificial Intelligence and Computer Science, Jiangnan University, Wuxi 214122, China; 4 School of Cyber Science and Engineering, Wuhan University, Wuhan 430072, China

**Keywords:** artificial intelligence, dairy products, quality assessment, multimodal sensing, explainable artificial intelligence

## Abstract

Artificial intelligence (AI) is transforming dairy quality assessment by enabling rapid, non-destructive, and data-driven monitoring across the dairy supply chain. Conventional analytical methods, although accurate, are often labor-intensive, time-consuming, and unsuitable for real-time applications. The integration of advanced sensing technologies with AI has therefore emerged as a promising approach for improving dairy quality control and food safety. This review examines recent advances in AI-enabled dairy quality assessment, covering spectroscopy-based sensing technologies, biomimetic sensing systems, machine vision, and multimodal data fusion. Particular attention is given to their applications in major dairy products, including raw milk, milk powder, cheese, and yogurt. Comparative analysis shows that AI-assisted sensing significantly enhances the accuracy, efficiency, and automation of compositional analysis, adulteration detection, microbial screening, freshness evaluation, and defect inspection, while multimodal approaches offer new opportunities for comprehensive quality assessment. The review further highlights the emerging role of explainable artificial intelligence (XAI) in improving the transparency and trustworthiness of dairy detection systems. Recent advances in feature attribution, decision interpretation, and uncertainty quantification are discussed, together with challenges related to data scarcity, model generalization, cross-instrument transferability, and explainability evaluation. Future developments in multimodal foundation models, standardized datasets, and explainable intelligent systems are expected to accelerate the deployment of trustworthy and scalable dairy quality assurance frameworks.

## 1. Introduction

Dairy products represent one of the most nutritionally significant food categories in the human diet [1], serving as a primary source of proteins, fats, lactose, and essential micronutrients that are critical for human growth, development, and immune function [2]. The quality and safety of dairy products are directly linked to public health and social stability [3]. However, due to the complex and variable composition of dairy products, their quality can be affected by environmental conditions, processing, and distribution stages; thus quality and safety issues exist throughout the entire supply chain [4]. This complexity not only increases the difficulty of risk identification but also places high demands on real-time, accurate, and traceable quality monitoring [5]. Consequently, the construction of efficient, full-chain quality detection systems has emerged as a critical challenge for the dairy industry.

Traditional methods for dairy quality assessment mainly rely on physicochemical analysis and microbiological testing [6]. For instance, fat and protein content are typically determined through chemical extraction, colorimetric assays, or chromatographic techniques [7]. Microbial safety is commonly evaluated through culture-based methods, including counting and identification [8]. Although these methods are considered gold standards and provide highly accurate quantitative results, they often require long testing times, complex sample preparation, and destructive sampling. Furthermore, some procedures rely on manual judgement, which may reduce consistency and stability [9]. To address these limitations, non-destructive detection methods based on advanced sensing technologies have attracted increasing attention [10]. Spectroscopic techniques, such as near-infrared (NIR) [11], Fourier-transform infrared (FTIR) [12], and Raman spectroscopy [13], can rapidly characterize dairy components through molecular vibrational information, facilitating efficient detection of fat, protein, and adulterants. Biomimetic sensing systems such as electronic nose (E-nose) [14] and electronic tongue (E-tongue) [15] digitize flavor and volatile compound detection by emulating human sensory perception. These methods offer distinct advantages in terms of rapidity, non-destructiveness, and online applicability. However, the high-dimensional and structurally complex data generated by these technologies necessitate advanced data modeling and pattern recognition approaches to transform raw signals into actionable decisions [16].

In recent years, artificial intelligence (AI) has been widely applied in dairy quality detection [17]. The field has evolved from traditional machine learning to deep learning and subsequently to multimodal approaches. Early studies used methods such as support vector machines (SVM) and random forests (RFs) for spectral data modeling to achieve compositional prediction and classification [18]. With the development of deep learning, convolutional neural networks (CNNs) have significantly improved the accuracy of image-based defect detection, while recurrent neural networks (RNNs) have demonstrated advantages in modeling time-dependent quality changes [19,20]. More recently, Transformer-based architectures have exhibited enhanced capabilities in processing spectral sequences and fusing multimodal data [21]. Attention mechanisms help identify critical wavelength regions influencing predictive outcomes.

Despite these advances, most studies focus on model accuracy and prediction performance, while giving limited attention to model transparency [22]. Deep learning models are frequently characterized as “black boxes” and their decision processes are difficult to interpret [23]. This limitation is a concern in highly regulated fields such as food safety. Regulatory agencies require detection results to be interpretable to support accountability and compliance auditing. At the same time, models without transparency are less likely to gain trust from industry and consumers [24]. Therefore, it is important to improve interpretability while maintaining high detection performance.

**Figure 1:**
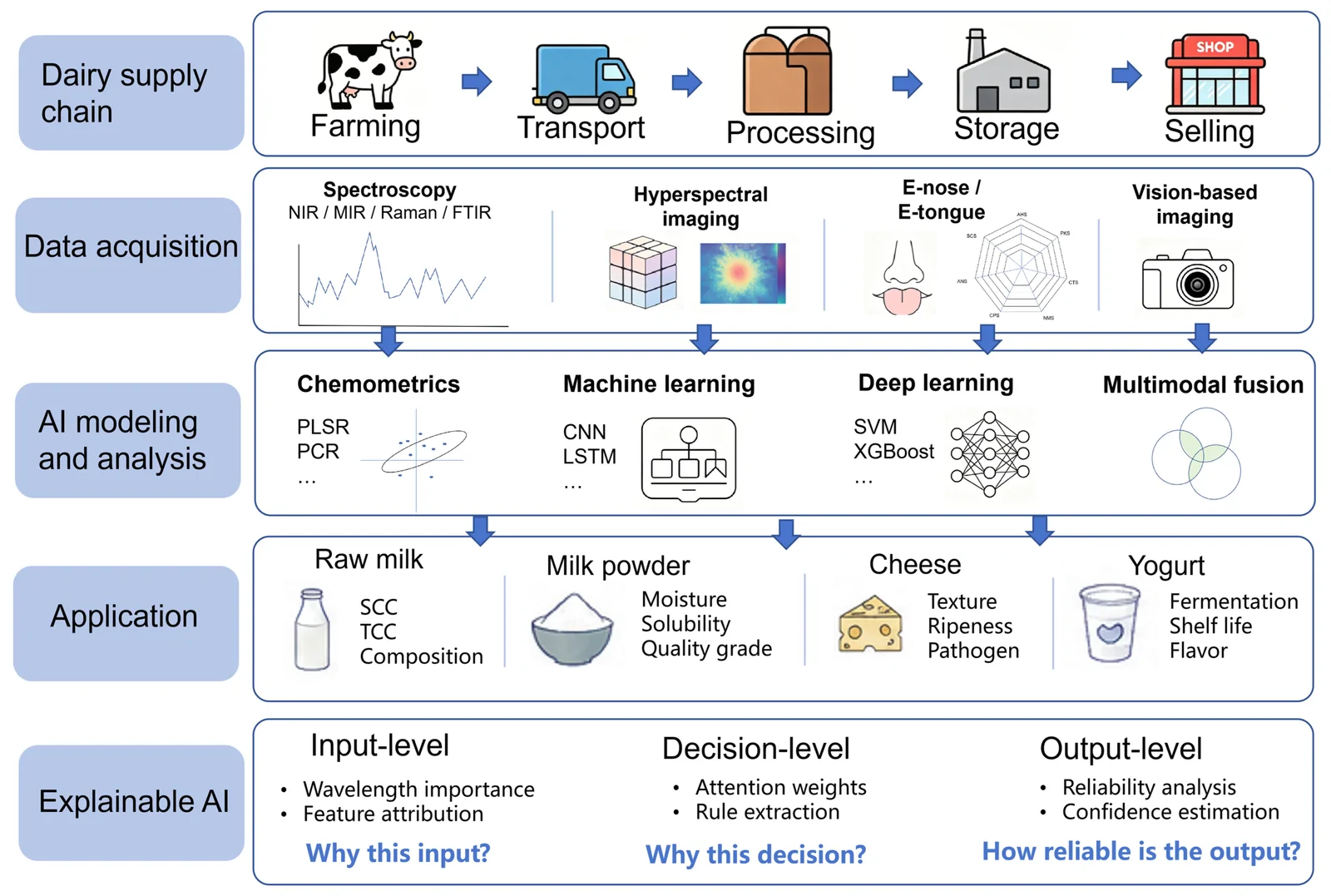
Overall framework of AI-driven dairy quality assessment.

The integration of these advanced sensing technologies with AI has attracted considerable research attention. Pérez Núñez et al. [25] reviewed AI applications across the dairy value chain, ranging from animal health and milk production to the processing and monitoring of dairy products, while also considering efficiency, sustainability, environmental impact, and product-demand forecasting. Erol et al. [26] provided a critical review of AI-enabled technologies under the Dairy 4.0 paradigm, covering precision livestock farming, in-line milk quality sensing, smart processing, and advanced ingredient development, with particular emphasis on model validation, generalizability, scalability, and real-world applicability. More specifically, Mhapsekar et al. [27] systematically reviewed the application of IoT and AI in milk quality analysis, focusing on composition monitoring, adulteration detection, microbial quality indicators, sensing technologies, data communication, and edge/cloud processing. Although these reviews provide valuable perspectives on AI in the dairy sector, they mainly emphasize either the broader dairy value chain, system-level Dairy 4.0 applications, or IoT-enabled milk quality monitoring. In contrast, as shown in Figure 1, this review focuses specifically on the convergence of advanced sensing technologies and AI for dairy quality assessment, integrating spectroscopy, biomimetic sensing, machine vision, multimodal data fusion, product-specific quality requirements, and explainable AI within a unified framework. This perspective enables a direct comparison of sensing modalities and AI strategies while highlighting their respective strengths, limitations, and remaining challenges for practical dairy quality assessment.

## 2. AI-Driven Sensing and Modeling Techniques for Dairy Quality Assessment

Quality detection of dairy products is essentially a data-driven multi-stage process. Its core procedures include data acquisition, signal preprocessing, feature modeling, and final quality determination. As sensing technology and computing power have advanced together, detection methods have evolved from single physicochemical detection to multi-source sensing fusion and gradually developed into a data-driven detection system centered on artificial intelligence. Based on differences in data sources and signal characteristics, current AI-enabled dairy detection technologies can be broadly categorized into spectroscopy and optical imaging technologies, bionic sensing technologies, and vision-based structural inspection technologies. Additionally, multimodal data fusion is becoming an important direction for improving detection performance.

### 2.1. Spectroscopy-Based Sensing and AI Integration

Spectroscopy captures absorption or scattering signals generated from the interaction between molecular vibrations and light. It enables rapid and non-destructive characterization of chemical components in dairy products. This technology covers a broad spectral range from near-infrared to Raman. Different spectral modalities vary in information content, sensitivity, and applicable scenarios. Consequently, their AI modeling approaches also differ significantly.

#### 2.1.1. NIR and Mid-Infrared Spectroscopy

NIR and mid-infrared (MIR) spectroscopy are widely used for quality and component analysis in dairy products. The fundamental principle lies in the selective absorption of specific wavelengths by molecular vibrations. Key components such as fat, protein, lactose, and moisture show distinct absorption features in corresponding bands. Therefore, quantitative determination and quality discrimination can be achieved through spectral inversion. In traditional studies, these methods mainly relied on chemometric models such as partial least squares regression (PLS), partial least squares discriminant analysis (PLS-DA), or principal component regression (PCR). These models established linear or weakly nonlinear relationships between spectral data and physicochemical indices to achieve prediction. Kang et al. [28] compared micro and benchtop NIR spectrometers to discriminate infant milk formula by storage time and predict particle size. FFT and wavelet denoising were applied to spectra, followed by PLS-DA and PLSR. The models achieved 89–100% correct identification and RPDPs of 2.29–2.98. They concluded that micro NIR with chemometrics is suitable as a PAT tool for IMF quality monitoring. However, chemometric methods require extensive spectral preprocessing. Their linear assumptions fail to capture complex nonlinear features. When sample matrices are complex or physical scattering interference exists, model accuracy and robustness often decrease significantly.

As data scale and complexity increased, early studies began to introduce machine learning methods such as SVM and RF. These methods enhanced the modeling capacity for nonlinear relationships. Buoio et al. [29] developed a rapid milk classification method combining a portable FT-NIR spectrometer with VC-SVM hybrid models without any pretreatment. The RBF kernel achieved perfect classification for whole and skimmed milk (100% sensitivity and specificity, MCC = 1). They concluded that this approach provides the dairy industry with a practical and efficient solution for rapid milk identification and fraud detection. These methods improved model generalization performance to some extent. They also achieved better results than traditional chemometric methods in dairy component prediction and adulteration identification.

In recent years, deep learning methods have gradually become mainstream. CNNs have been used to automatically extract features directly from raw spectra. This avoids the limitations of manual feature engineering. One-dimensional convolutional networks (1D-CNNs) have demonstrated high efficiency in spectral sequence modeling. Chen et al. [30] proposed an MBML Network integrating multi-branched and multi-layered features for NIRS quantitative analysis. The model automatically extracts features from raw spectra without preprocessing and fuses shallow and deep spectral characteristics. Evaluated on multiple datasets including milk, it consistently outperformed PLS, SVR, Principal Component Analysis (PCA) and Artificial Neural Networks (ANNs). They concluded that MBML Net achieves superior prediction accuracy and versatility while significantly simplifying quantitative analysis workflows. Meanwhile, recurrent neural network models such as long short-term memory (LSTM) introduce advanced sequence learning mechanisms. They can effectively process spectral sequential data and capture key wavelengths that strongly influence prediction results. This further improves modeling accuracy and stability. Li et al. [31] presented a rapid quantitative approach using NIR transmission spectroscopy to detect psychrotrophic bacteria in raw milk. They developed PLSR, RF, and LSTM models after spectral preprocessing and feature selection. The SG-UVE-LSTM model achieved the best performance with an R^2^*p* of 0.9277 and RPD of 3.8256. They concluded that this method offers a rapid, efficient, and low-cost solution for microbial monitoring in dairy quality and safety control.

#### 2.1.2. Raman Spectroscopy

Raman spectroscopy detects inelastic scattering signals arising from molecular vibrations, generating unique molecular fingerprints that reveal detailed chemical structure information. In dairy quality control, it is particularly suited for authenticity verification, brand discrimination, and trace adulterant detection, where specific functional groups must be identified. Its sharp spectral peaks with minimal band overlap offer superior resolution compared to infrared spectroscopy for distinguishing minor chemical differences. However, Raman signals are inherently weak and easily overwhelmed by fluorescence background and noise, making intelligent data processing indispensable for practical dairy applications.

Brand authentication and origin tracing of dairy products remain challenging because compositional differences between brands are often subtle. Raman spectroscopy can capture minor variations in lipid and protein vibrational structures, yet the resulting high-dimensional spectra contain substantial redundant information. Machine learning-based feature selection and pattern recognition are therefore required to extract discriminative signals and achieve reliable classification. Li et al. [32] investigated discriminative Raman features among three dairy brands using SVM, ELM, and CNN algorithms. They found that fusing intervals such as 890–980 cm^−1^ and 1410–1500 cm^−1^ achieved 100% recognition accuracy, with ELM completing classification in under 0.3 s and CNN in about 80 s. This demonstrates that algorithm-specific feature interval selection combined with machine learning enables efficient and accurate brand-level discrimination.

Powdered milk adulteration poses another serious threat to dairy safety; although Raman spectroscopy reflects changes in protein and fat vibrations caused by such mixing, spectral overlap and scattering interference from the powder matrix complicate direct quantification. Robust chemometric modeling coupled with variable selection is needed to build accurate quantitative relationships. Li et al. [33] employed Raman spectroscopy combined with deep learning for rapid, non-destructive detection of buffalo milk adulteration. Multiplicative Scatter Correction (MSC) preprocessing enhanced PLS-DA classification accuracy to 100% for qualitative analysis, while MSC-CNN regression models achieved high quantitative prediction (R^2^ > 0.93) for sodium bicarbonate and sodium citrate with low detection limits, offering an effective solution for milk quality control. Ait El Alia et al. [34] applied Raman spectroscopy with chemometric techniques to detect cow milk powder adulteration in camel milk powder. PCA captured 99.6% spectral variance, while optimized PLSR models achieved near-perfect prediction (R^2^test = 99.94%, RMSEtest = 1.10%). The integration of artificial intelligence with Raman spectroscopy effectively overcomes the technique’s inherent weaknesses of weak signal intensity and strong background interference, offering a rapid and non-destructive means for dairy authenticity verification and adulteration detection.

#### 2.1.3. Hyperspectral Imaging (HSI)

Hyperspectral imaging technology integrates two-dimensional spatial information and one-dimensional spectral information, enabling pixel-level analysis of dairy product samples. Its data format is typically a three-dimensional data cube (spatial × spatial × wavelength), which provides rich features but also introduces challenges of computational complexity and information redundancy. In the context of dairy quality assessment, AI-driven methods have been increasingly employed to decode these high-dimensional data, facilitating applications ranging from compositional quantification and adulteration detection to cross-domain adaptation in milk and milk powder.

For compositional quantification, Xu et al. [19] proposed a joint learning-based selector and predictor (JLSP) for milk fat content prediction using HSI. The model employs an actor–critic algorithm inspired by reinforcement learning to optimize feature selection, achieving an R^2^ of 0.9734 and an MSE of 0.0573. This study demonstrates that deep learning effectively captures nonlinear spectral–compositional relationships, providing an interpretable approach for milk quality assessment.

In the field of dairy adulteration detection, Aqeel et al. [35] developed a nondestructive HSI pipeline combined with machine learning for milk adulterant detection. Among tested algorithms, linear discriminant analysis excelled by learning spectral signatures, attaining 100% validation accuracy. This work establishes that HSI coupled with discriminant analysis offers a practical solution for detecting and categorizing milk adulteration. While the preceding studies address known adulteration types, multi-class contaminant detection presents greater analytical complexity. Iqbal et al. [36] introduced a hybrid ensemble learning framework integrating gradient boosting, XGBoost, LightGBM, and multilayer perceptron for noninvasive milk contaminant classification. The approach achieved 96% validation accuracy, significantly outperforming existing methods. Such ensemble strategies demonstrate that combining multiple AI models enhances robustness in real-time quality assurance.

However, the above approaches assume consistent sample distributions between training and testing, which rarely holds in practical deployment. To address domain shift, Ruan et al. [37] employed metric-based meta-learning with HSI for camel milk powder adulteration detection under domain-shifted conditions. Using cow-adulterated samples for training and goat-adulterated samples for testing, the method achieved 84.4% comprehensive accuracy, substantially surpassing conventional classifiers. This highlights meta-learning’s potential in cross-domain scenarios with limited labeled data.

Overall, spectroscopy-based sensing provides a strong foundation for AI-driven dairy quality assessment because it offers rapid and non-destructive access to compositional and molecular information. However, the suitability of a given AI strategy depends strongly on the characteristics of the spectral data and the intended application. Conventional chemometric models remain advantageous for relatively small datasets and applications requiring straightforward interpretation, whereas machine-learning and deep-learning approaches provide greater flexibility for nonlinear and high-dimensional spectral relationships. Nevertheless, the reported advantages of more complex models cannot be interpreted independently of dataset size, preprocessing procedures, instrument configuration, and validation strategy. A major limitation across the literature is that many models are evaluated under controlled laboratory conditions, making their robustness to changes in sample composition, instruments, and production environments difficult to establish.

Generative AI is beginning to provide new strategies for addressing the limited availability of labeled spectral data in dairy quality assessment. Generative adversarial networks (GANs) have been used to synthesize additional spectra to alleviate small-sample limitations and reduce overfitting, while transformer-based generative models provide a potential route for learning more complex spectral distributions [21,38]. These approaches suggest that generative models can contribute not only to data augmentation but also to representation learning and model transfer. However, synthetic spectra must remain physically and chemically plausible, and improvements obtained through augmentation require validation using independent real-world samples. Therefore, generative AI should currently be regarded as a complementary strategy for addressing data scarcity rather than a replacement for high-quality experimental data. Future studies should therefore place greater emphasis on external validation, cross-instrument transfer, uncertainty estimation, and standardized evaluation protocols.

### 2.2. E-Nose and E-Tongue Systems

E-nose and E-tongue systems utilize sensor arrays to mimic human olfactory and gustatory functions, enabling the rapid detection of volatile organic compounds and dissolved components in dairy products. These bionic sensing technologies have demonstrated significant potential in freshness evaluation, flavor profiling, and spoilage detection. Advanced machine learning algorithms, including PCA, Linear Discriminant Analysis (LDA), SVM, and ANN, are routinely integrated with these sensor systems to enhance pattern recognition accuracy and classification performance in complex dairy matrices [39]. Compared with conventional chromatographic or mass spectrometric methods, this integrated approach offers distinct advantages of low cost, rapid response, simple operation, and minimal sample preparation, making it particularly suitable for online monitoring and on-site screening applications.

Recent studies have demonstrated the feasibility of e-nose systems for dairy adulteration screening across different products and adulteration scenarios, although the reported performance has been evaluated under study-specific experimental conditions. Zhang et al. [40] developed a rapid, non-invasive detection method for yak milk adulterated with water, Holstein milk, and cattle-yak milk using an e-nose coupled with machine learning algorithms and multivariate statistical analysis. Their results indicated that both PCA and canonical discriminant analysis (CDA) effectively differentiated adulterated samples, with the random forest model achieving an overall area under the curve (AUC) exceeding 0.99 and perfect classification (AUC = 1.00) for water-adulterated groups. The multilayer perceptron (MLP) model attained average accuracies above 96% across all adulteration categories, reaching 99.8% for Holstein milk-adulterated samples, thereby establishing a robust, low-cost analytical solution for on-site market surveillance of high-value specialty dairy products. Darvishi et al. [41] employed an e-nose equipped with eight metal oxide sensors to detect melamine adulteration in milk powder at concentrations ranging from 1 to 5 ppm in both dry and wet forms. Principal Component Analysis, discriminant analysis (DA), and Support Vector Machine methods were utilized for sensor response pattern analysis and classification. Quantitative Discriminant Analysis (QDA) achieved a precision of 99.5%, while Multi-Discriminant Analysis (MDA) reached 98.5%, demonstrating that e-nose technology combined with chemometric methods constitutes an effective tool for rapid detection and classification of adulterated milk powder.

E-tongue technologies are specifically suited for analyzing non-volatile dissolved constituents and taste-active compounds in the liquid phase, thereby complementing e-nose systems and enabling comprehensive assessment of dairy authenticity and sensory properties. Perez-Gonzalez et al. [42] developed a potentiometric e-tongue for rapid milk adulteration detection. The system discriminated raw, pasteurized, and goat milk, and identified water and cow milk adulterants in goat milk. PCA and PLS regression correlated sensor responses with fat, protein, and lactose content, achieving R^2^ values up to 0.97 during validation. Huang et al. [15] proposed an e-tongue approach coupled with sensory evaluation and chemical analysis to decode dairy flavor profiles. PCA effectively discriminated milk and yogurt samples based on processing and compositional variables, while PLSR models reliably predicted sensory attributes (R^2^Y = 0.971–0.972).

Collectively, these studies confirm the strong potential of e-nose and e-tongue systems as rapid, low-cost, and non-invasive tools for dairy authenticity screening and flavor profiling. However, several technology-specific challenges remain that distinguish these bionic sensing systems from other optical or imaging modalities. First, sensor array cross-sensitivity and temporal drift constitute a fundamental hardware limitation, the non-specific response of metal oxide or potentiometric sensors often leads to overlapping signals from different analytes, and sensor baseline shifts over time complicate long-term deployment without frequent recalibration. Second, sample matrix effects, particularly variations in temperature, pH, and fat content, can substantially alter sensor responses, and unlike spectroscopic techniques where chemometric preprocessing can partially compensate for scatter effects, these matrix-induced signal changes in e-nose/e-tongue data are often non-linear and less tractable with standard correction methods. Third, the interpretability of bionic sensor data faces unique difficulties: while spectroscopic features can be traced to specific molecular vibrations, the collective sensor array outputs encode complex, overlapping physicochemical interactions, making it harder to establish direct cause–effect relationships between individual sensor signals and specific flavor compounds. Furthermore, the studies reviewed here predominantly relied on linear models (PCA, PLSR, LDA) under controlled laboratory conditions; the potential of more advanced non-linear architectures, such as deep neural networks, for enhancing robustness and generalization in real-world dairy processing environments remains underexplored. Addressing these challenges will require the development of more robust sensor materials, standardized calibration protocols, and hybrid modeling strategies that combine mechanistic knowledge with data-driven learning to improve both the reliability and interpretability of bionic sensing systems for industrial dairy quality control.

### 2.3. Machine Vision and Image-Based Inspection

Machine vision technology acquires surface or internal image information of dairy products through optical imaging systems, enabling automated evaluation of color, texture, morphology, and structural defects. Unlike spectroscopy, which focuses on chemical component analysis, visual inspection targets product appearance quality and physical integrity, serving as an essential tool for dairy product appearance grading, foreign object removal, and packaging integrity inspection. Early approaches relied on manual feature extraction techniques, such as color histograms and texture descriptors, combined with conventional classifiers. However, these methods exhibited limited adaptability to complex scenarios. With advances in imaging hardware and computational algorithms, the field has evolved from rule-based image processing to intelligent visual analysis based on deep learning, demonstrating enhanced capability in detecting surface quality issues including mold contamination on cheese, texture abnormalities, and color variations.

The emergence of deep learning has greatly changed the field of dairy visual inspection. CNNs automatically extract hierarchical visual features from raw pixels through end-to-end learning, substantially outperforming traditional methods in classification tasks such as cheese mold recognition and yogurt surface texture grading. Zedda et al. [43] proposed the first public dataset of cheese wheel images depicting various products at distinct ripening stages; through a lightweight hierarchical feature aggregation strategy that fuses deep features from EfficientNet and DarkNet-53, their framework simultaneously classifies cheese types and ripeness levels, achieving an F-measure of 0.991. This work demonstrates the potential of integrating multi-scale deep representations for hierarchical quality grading in dairy products. Pérez-Calabuig et al. [44] used ResNet34 to detect melamine adulteration and monitor yogurt shelf life, by training CNNs on images of natural, sweetened, and lemon-flavored yogurts captured under two shutter speeds (1/30 s and 1/250 s); they achieved accuracies of 94.30% and 91.74% for melamine detection, and 96.95% and 96.86% for freshness classification, respectively. These results indicate that CNN-based image analysis is applicable as a rapid, non-invasive, and cost-effective tool for preliminary screening in dairy quality assurance.

When detection requirements shifted from “whether defects exist” to “where defects are located”, object detection models such as YOLO and the Faster R-CNN series were introduced, achieving real-time localization and category determination of defect positions to satisfy the time constraints of high-speed sorting on production lines. Konstantinidis et al. [45] proposed a real-time dairy product tracking method for Dairy 4.0 production lines and introduced the YogDATA dataset, comprising yogurt cup images with LOT numbers captured on an industrial production line. They evaluated Mask R-CNN and YOLO v5.0 for the automated recognition and localization of yogurt cups, reporting similar performance with approximately 99% precision. This work demonstrates the applicability of object detection architectures in automating packaging procedures and quality-related tracking within industrial dairy environments.

The evidence reviewed in this section indicates that machine vision is particularly effective for quality attributes that can be expressed through visible or spatially localized features, including color, surface defects, ripeness, packaging integrity, and product tracking. CNN-based classification is suitable for identifying predefined defect categories, whereas object-detection architectures are more appropriate when defect localization and real-time inspection are required. However, visual models have inherent limitations when quality changes are not manifested as observable surface features or when chemical composition must be inferred from indirect image patterns. Their performance may also be sensitive to illumination, camera configuration, background conditions, and packaging variability. Thus, machine vision should be regarded as complementary to spectroscopic and sensor-based approaches rather than as a universal replacement, and future research should emphasize environmental robustness, external validation, and multimodal integration.

### 2.4. Multimodal Data Fusion in Dairy Detection

While individual technologies excel in specific tasks, single-modality information acquisition ultimately has inherent limitations. Spectroscopy excels at chemical component analysis but lacks spatial details; visual imaging captures surface defects yet fails to reveal internal chemical changes; electronic nose and tongue systems reflect overall flavor characteristics but cannot locate the source of problems. Multimodal data fusion integrates heterogeneous or complementary sensing information to build a more comprehensive and robust quality characterization system, which has become an important development direction for intelligent dairy detection.

Recent advances in sensing chemistry have enabled integrated multimodal transduction platforms that generate multiple physicochemical readouts within a single sensing system. For instance, Zhang et al. [46] developed a coumarin–benzothiazole probe and kanamycin aptamer-based multimodal aptasensing platform (DR1/K-apt) for label-free detection of kanamycin in milk samples. The proposed system simultaneously generated colorimetric, fluorescent, and visual signals through competitive binding interactions, achieving a sensitive detection limit of 0.001 mM within 30 min. Notably, the fluorescence signal exhibited distinct green–red–green color transitions, enabling the construction of a smartphone-based portable paper device (K-cPAD) for instrument-free on-site quantification. This work demonstrated that multimodal signal transduction could effectively overcome the limitations of single-readout methods and provide cross-validated detection performance in complex food matrices.

With the rapid development of artificial intelligence, deep learning has enabled higher-level semantic fusion of multimodal data. Attention mechanisms and deep neural networks can dynamically weight or jointly encode complementary features from diverse sensors, yielding more robust and adaptive prediction models than conventional static fusion strategies. Li et al. [47] developed a multimodal deep learning system for non-destructive chili paste quality evaluation fusing hyperspectral imaging, near-infrared spectroscopy, and physicochemical data. Multiplicative Scatter Correction and Uninformative Variable Elimination optimized feature extraction. A CNN-LSTM model with Mixup augmentation achieved an R^2^ of 0.9554–0.9826, establishing the first framework for chili paste fermentation. Such advanced fusion frameworks have achieved substantial success in quality assessment of various foods.

Multimodal sensing offers an important conceptual advantage because different modalities provide complementary information that cannot be captured by a single sensing system. Spectroscopy can characterize chemical composition, machine vision can provide spatial and morphological information, and e-nose/e-tongue systems can capture volatile and sensory-related characteristics. Evidence from dairy and other food matrices suggests that multimodal AI can potentially improve the completeness and robustness of quality assessment. However, multimodal dairy studies remain relatively limited compared with single-modality applications, and the reported benefits cannot yet be considered broadly established. Major challenges include synchronization and calibration of heterogeneous sensors, feature alignment, missing data, increased computational complexity, and the need for sufficiently large multimodal datasets. Notably, the application of deep learning-based multimodal approaches in dairy detection remains scarce, even though implementations in other food matrices have demonstrated the feasibility of this strategy. Therefore, future research should prioritize standardized multimodal datasets, independent validation, and interpretable fusion strategies capable of linking complementary sensor signals to specific quality risks, while integrating artificial intelligence with multimodal data fusion to achieve more comprehensive and intelligent dairy quality assessment.

## 3. AI-Driven Quality Assessment Across Dairy Product Categories

**Figure 2:**
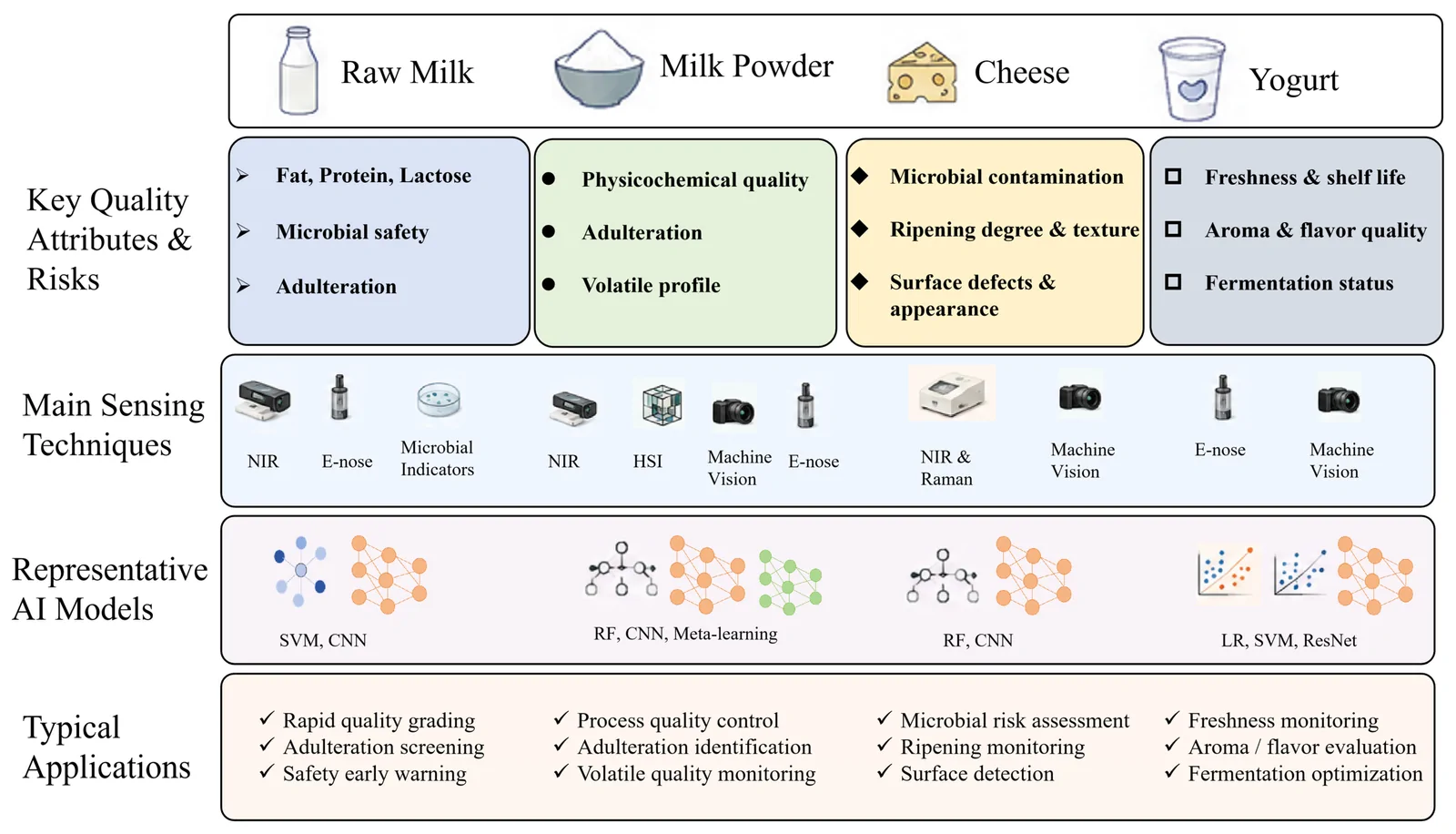
AI-driven assessment strategies for different dairy product categories.

Quality risks in dairy products are closely associated with processing technologies, and the hazard factors faced by different products vary considerably. As shown in Figure 2, raw milk serves as the source of all dairy products. Its compositional variation and microbial control status directly determine the quality foundation of subsequent products. Although microbial growth is inhibited in milk powder after drying, adulteration and foreign matter contamination are more likely to occur. The long fermentation and ripening process of cheese creates complex conditions for mold growth and quality variation. Yogurt contains active bacterial cultures and exhibits a gel structure, making it highly sensitive to fermentation degree and storage conditions. These differences dictate that targeted detection methods must be adopted for different dairy products. Artificial intelligence offers flexibility, enabling customized detection schemes to be designed according to the characteristics of each product. This chapter reviews the quality risk profiles of four core dairy categories: raw milk, milk powder, cheese, and yogurt. The applicable limitations of traditional detection methods are analyzed, and the ways in which sensing technologies and intelligent algorithms can build customized detection solutions for the key quality attributes of each product are further explained.

### 3.1. Raw Milk Quality Grading and Safety Screening

Raw milk is the origin of the dairy industry. Its quality is defined by three dimensions: compositional indices (fat, protein, lactose, and total solids), hygiene indices focusing on somatic cell count (SCC) and total bacterial count (TBC), and authenticity indices (adulteration practices such as water dilution, melamine addition, and blending with cheap fillers). These categories are interrelated: compositional anomalies may indicate adulteration, while hygiene deterioration accelerates compositional degradation.

Traditional detection systems rely on specialized laboratory instrumentation. SCC determination relies on direct microscopic counting or flow cytometry; the former is labor-intensive and operator-dependent, whereas the latter achieves high throughput but demands prohibitive investment and maintenance costs. TBC assessment depends on plate counting with a 24–72 h incubation period, which cannot satisfy real-time procurement decisions, or on ATP bioluminescence, which offers rapid on-site testing but suffers from complex sample preparation and inferior quantitative accuracy. Adulteration detection remains particularly challenging because classic methods such as the Kjeldahl approach cannot distinguish protein nitrogen from non-protein nitrogen.

An integrated solution for full-dimensional quality screening of raw milk is provided by the integration of artificial intelligence and multi-source sensing technologies. For compositional prediction, rapid quantification of fat, protein, and lactose has been achieved by near-infrared spectroscopy combined with partial least squares regression or support vector regression. Diaz-Olivares et al. [48] developed a fiber-optic spatially resolved near-infrared spectroscopy system for in-line raw milk analysis, using partial least squares regression to simultaneously predict fat, protein, and lactose with RMSEP values below 0.10%, 0.13%, and 0.13% (*wt*/*wt*), respectively, by optimizing illumination-to-detection fiber distances. For integrated quality grading incorporating compositional and hygiene indices, Yang et al. [49] proposed a hybrid variable selection method combining XGBoost-based forward feature selection with genetic algorithm optimization, and constructed a support vector machine model for raw milk quality grade classification based on fat, protein, and somatic cell count, achieving 94.84% prediction accuracy and a 94.21% F1 score. For adulteration identification, strong potential has been shown by the combination of near-infrared spectroscopy with machine learning classifiers. Wei et al. [50] employed near-infrared spectroscopy coupled with CNN to simultaneously classify and quantify adulteration in specialty milks. The model differentiated camel, donkey, and horse milk from cow milk with perfect classification accuracy, and gradient-weighted class activation mapping visualization revealed the distinct spectral regions driving classification decisions. For quantitative adulteration analysis across a 2–80% cow milk concentration range, the convolutional neural network outperformed partial least squares regression models with R^2^pre exceeding 0.9995 and a ratio of performance to deviation above 47.91.

For raw milk, the reviewed evidence indicates that AI-enabled sensing can address multiple quality dimensions, including composition, hygiene-related indicators, and adulteration, but no single sensing modality is sufficient for complete quality characterization. NIR-based approaches are particularly suitable for rapid compositional prediction, while machine-learning and deep-learning models can extend the capability toward integrated grading and authenticity assessment. However, compositional prediction and adulteration classification represent different analytical tasks and should not be evaluated using the same performance criteria. In addition, microbial and hygiene-related risks remain more difficult to characterize non-destructively than major compositional parameters. The current evidence is also dominated by controlled datasets, indicating a need for larger field datasets, external validation, and integration of complementary sensing information to achieve reliable full-dimensional raw milk quality assessment.

### 3.2. Milk Powder Processing Quality and Adulteration Identification

Milk powder is the concentrated form of liquid milk. The spray drying process constitutes the core stage of milk powder production. Minor fluctuations in inlet air temperature, outlet air temperature, or total solids content of concentrated milk can cause excessive residual moisture or protein heat damage. Excessive moisture triggers Maillard reactions and lipid oxidation, shortening shelf life and producing scorched flavors. Overheating induces whey protein denaturation and lysine loss, reducing nutritional value. Solubility is a key functional attribute of milk powder, jointly influenced by particle density, surface free fat content, and the degree of protein thermal aggregation. Traditional solubility index determination is time-consuming and exhibits poor repeatability.

Traditional milk powder quality control relies on offline laboratory analysis. For example, adulteration detection is performed using high-performance liquid chromatography, enzyme-linked immunosorbent assays, or mass spectrometry. Although precise, these methods involve complex sample pretreatment, expensive instrumentation, and limited throughput. They are more suitable for confirmatory testing than for rapid screening during raw material intake or finished product release. For physical contaminants, X-ray inspection machines have been deployed on some production lines. However, traditional algorithms are based on density thresholds and morphological rules. They show poor adaptability to low-density foreign objects and complex packaging backgrounds.

With the integration of artificial intelligence and advanced sensing technologies, spectroscopic and imaging methods have been increasingly employed for milk powder quality control. Near-infrared spectroscopy combined with chemometric algorithms enables rapid quantification of key physicochemical indicators. Peng et al. [51] developed a miniaturized near-infrared spectroscopic system coupled with random forest regression for simultaneous prediction of protein, fat, moisture, and acidity in yak milk powder, achieving correlation coefficients of 0.9846, 0.9642, 0.9915, and 0.9819, respectively, and ratio of performance to deviation values of 5.4242, 5.4697, 20.1447, and 14.1533, demonstrating the feasibility of portable devices for rapid production-line quality monitoring. Hyperspectral imaging extends spectral analysis by incorporating spatial information, offering unique advantages for detecting subtle compositional variations and cross-variety adulteration. Ruan et al. [37] employed hyperspectral imaging with a prototypical network meta-learning framework to detect camel milk powder adulterated with cow milk powder and generalized the model to unseen goat milk powder adulteration, achieving 98.92% accuracy for pure samples and 77.69% accuracy for challenging 70% adulteration levels under domain-shifted, few-shot conditions, substantially outperforming support vector machine, backpropagation, and standard convolutional neural network approaches. Beyond spectroscopic techniques, deep convolutional neural networks applied to conventional photographic images provide cost-effective tools for screening chemical adulterants. Pradana-López et al. [52] trained a ResNet34 architecture on 3100 images of three powdered milk brands to classify 31 groups spanning trace melamine concentrations down to 0.5 ppm, attaining 98.7% validation accuracy and a 3.0% blind-test misclassification rate, proving its effectiveness as a rapid safety control method.

Electronic nose technology indirectly reflects the degree of lipid oxidation and protein denaturation by monitoring changes in volatile flavor compounds of milk powder. Darvishi et al. [41] deployed an electronic nose with eight metal oxide sensors to capture volatile patterns of pure and melamine-adulterated milk powder across five concentration levels in both dry and wet forms, employing support vector machine, quadratic discriminant analysis, and multi-discriminant analysis for pattern recognition, achieving classification precision up to 99.5% and validating machine-learning-driven electronic nose systems for rapid adulteration screening.

Milk powder quality assessment requires a combination of chemical, physical, and volatile-related information, and the reviewed studies illustrate the complementary value of different sensing–AI strategies. NIR provides rapid information on physicochemical attributes, HSI offers additional spatial–spectral information for adulteration detection, machine vision provides a relatively low-cost route for screening visually or indirectly expressed contaminants, and E-nose systems can capture changes in volatile profiles. However, these approaches are not directly interchangeable because they target different quality attributes and operate at different levels of information. In particular, the domain-shift results reported for HSI indicate that strong performance on controlled datasets may decline substantially when the adulterant or sample distribution changes. Future research should therefore prioritize integrated evaluation under realistic processing and storage conditions, together with cross-product validation and standardized benchmarks.

### 3.3. Cheese Quality Assessment and Defect Detection

Cheese manufacturing involves multiple stages where chemical composition and microstructure continuously evolve, introducing quality risks throughout the process. Surface mold contamination by Penicillium and Aspergillus during ripening represents the most visible defect, compromising appearance and potentially producing mycotoxins. Physical defects such as cracks and bulges arise from uneven acidification, incomplete whey drainage, or improper temperature and humidity control. Textural issues including excessive proteolysis from over-ripening or insufficient protein network degradation from under-ripening result in powderiness, oil exudation, or overly firm matrices. Additionally, foreign matter contamination and fat content discrepancies remain critical concerns. Conventional quality control relies on visual inspection for surface mold, which is inefficient and prone to missing early colonies. Texture analysis and chemical composition determination require destructive sampling and off-line laboratory procedures, preventing whole-wheel assessment and compromising product integrity.

Meenakshi et al. [53] systematically summarized the application of non-destructive testing technologies in cheese quality evaluation. Spectroscopic methods including near-infrared, Fourier-transform infrared, fluorescence, and surface-enhanced Raman spectroscopy have been widely explored for microbial and compositional analysis. Hyperspectral imaging provides both spatial and spectral information for detecting surface and subsurface defects. Electronic nose systems monitor volatile organic compounds to assess spoilage and ripening status. The integration of artificial intelligence with these sensing modalities represents a significant advancement, enabling automated feature extraction, pattern recognition, and predictive modeling that overcome the limitations of conventional chemometric approaches.

In the domain of pathogen detection, the combination of near-infrared spectroscopy with machine learning and deep learning algorithms has demonstrated substantial potential for rapid microbial screening. Meenakshi et al. [38] employed NIR spectroscopy coupled with Random Forest, Support Vector Machine, and one-dimensional Convolutional Neural Network classifiers to simultaneously classify Listeria monocytogenes contamination levels and differentiate strains in fresh cheese. To address the inherent limitation of limited spectral data availability, a conditional Wasserstein generative adversarial network with gradient penalty was implemented for spectral augmentation. The incorporation of synthetic spectra substantially enhanced model generalization and reduced overfitting, with SVM achieving a classification accuracy of 95.7% and 1D CNN attaining 95.6% on the augmented dataset. These results confirm that GAN-assisted spectral augmentation combined with NIR spectroscopy constitutes a feasible real-time screening approach for early Listeria detection in cheese production environments.

For automated ripeness assessment and surface quality evaluation, computer vision approaches provide non-invasive alternatives to subjective human inspection. Perniciano et al. [54] developed the first comprehensive public dataset of cheese wheel images spanning multiple products and ripening stages, and subsequently proposed an AI-based framework designated CRDet. This framework employs a Random Forest classifier operating on deep features extracted from a pre-trained DenseNet-201 architecture, with dimensionality reduction achieved through correlation-based feature selection. Notably, the approach achieves F1-scores exceeding 90% across diverse cheese subsets without requiring network fine-tuning, relying exclusively on off-the-shelf methods. The robust cross-variety performance demonstrates that CRDet effectively overcomes the traditional dependence on human expertise for ripeness evaluation, offering an accessible and efficient automated solution for industrial cheese quality control.

Cheese quality assessment represents a particularly challenging application because quality changes involve both internal biochemical processes and externally observable structural and visual characteristics. The reviewed studies indicate that spectroscopic and AI-based approaches are promising for rapid microbial or compositional screening, whereas computer vision is especially useful for ripeness and surface-quality evaluation. These modalities therefore address complementary aspects of cheese quality rather than competing for a single optimal solution. Nevertheless, the available studies remain limited by differences in cheese varieties, ripening conditions, imaging environments, and microbial targets, which restrict direct comparison across studies. Further research should emphasize multi-variety validation, longitudinal monitoring over the ripening process, and multimodal approaches capable of jointly representing biochemical, sensory, and visual quality changes.

### 3.4. Yogurt Freshness Monitoring and Fermentation Optimization

Yogurt is a dairy product characterized by active bacterial fermentation. Its quality is formed during fermentation and continuously declines during storage and transportation. The aroma profile is directly determined by accurate identification of the fermentation endpoint. Insufficient fermentation leads to underdeveloped flavor, while excessive fermentation results in unbalanced aroma dominated by harsh off-notes. During storage and transportation, temperature fluctuations accelerate quality degradation and alter volatile compound composition, compromising sensory acceptability. The diversity of yogurt aroma types, commonly described as fermented, cheesy, milky, and fruity, has not been systematically established, making objective classification difficult. Meanwhile, sensory evaluation suffers from perceptual ambiguity between similar aroma categories and cannot be automated for high-throughput screening. In addition, adulteration with substances such as melamine and the progressive loss of freshness during shelf life further threaten product integrity and consumer safety. Traditional yogurt quality control relies on offline sampling. pH meters and titratable acidity measurement disrupt the sealed environment of fermentation tanks and limit measurement frequency. Sensory evaluation depends on expert experience and cannot quantify viable bacterial decline. Conventional gas chromatography–mass spectrometry (GC–MS) for aroma analysis is time-consuming and requires specialized personnel, while chemical reagent-based methods for adulteration detection are destructive and inefficient for routine quality assurance.

Recent studies have explored AI-enabled electronic-nose and related chemical-feature approaches for yogurt aroma classification, with reported results ranging from sensor-array-based pattern recognition to interpretable models based on volatile organic compound (VOC) profiles and aroma-active compounds. Zeng et al. [55] employed quantitative descriptive analysis to classify commercial plain yogurt aromas into fermented, cheesy, milky, and fruity types, and developed SVM-based e-nose models achieving 0.933 accuracy; reduced sensor datasets still reached 0.867 accuracy and 0.872 F1 with logistic regression. The response patterns of metal oxide sensor arrays are processed using machine-learning algorithms to discriminate VOC profiles associated with sensory-defined aroma categories. These results indicate that e-nose sensor response patterns can be associated with sensory-defined aroma categories, although classification performance remains dependent on sensor selection and dataset composition. Operators can infer yogurt aroma profiles without relying on human sensory panels. Furthermore, refined smaller-size e-nose datasets maintain satisfactory classification performance while reducing hardware complexity. This provides a theoretical basis for rapid plain yogurt aroma type identification.

Complementary approaches based on aroma-active compound concentrations provide a more chemically interpretable alternative to sensor-array pattern recognition. Qiu et al. [56] constructed interpretable logistic regression models using aroma-active compound concentrations, achieving AUC ROC > 0.8 for yogurt aroma type prediction, and identified ten indicator compounds via feature importance analysis to establish explicit classification criteria. Logistic regression uses linear decision boundaries to separate different aroma types, yielding straightforward and chemically interpretable classification rules. Indicator compounds such as hexanoic acid, decalactone, and acetoin quantitatively characterize yogurt sensory properties. This achieves automated and reproducible aroma type prediction without relying on expert panels, providing a practical pipeline for monitoring yogurt sensory attributes.

For quality control and shelf-life monitoring, AI-driven optical methodologies combined with convolutional neural networks capture surface features and color changes of yogurt through high-resolution cameras under controlled lighting conditions. Pérez-Calabuig et al. [44] developed a ResNet34-based optical methodology under two lighting conditions, achieving 94.30% and 91.74% accuracy for melamine detection and 96.95% and 96.86% for freshness classification, with per-class metrics exceeding 0.90 in over 85% of classes. ResNet34 architectures are particularly suitable for extracting deep visual features from photographic data to detect adulteration and classify freshness states. By learning discriminative image patterns, melamine contamination and quality degradation can be identified non-invasively without chemical reagents or specialized personnel. This provides a rapid, cost-effective preliminary screening tool to support food safety and quality assurance in the dairy sector.

The reviewed studies indicate that yogurt quality assessment benefits from combining sensory-related, physicochemical, and visual information. E-nose and flavoromics-based models are suitable for characterizing aroma profiles and providing more objective sensory classification, whereas image-based models offer rapid screening of freshness and adulteration-related changes. The main strength of these approaches is their non-destructive and potentially high-throughput nature; however, their predictions remain closely tied to the specific yogurt formulations, aroma categories, storage conditions, and imaging environments used during model development. In addition, aroma classification based on sensor responses or selected aroma-active compounds does not fully capture the dynamic biochemical processes occurring during fermentation and storage. Future studies should therefore integrate temporal process variables with multimodal sensing data and evaluate model transferability across formulations, batches, and storage conditions.

### 3.5. Comparative Mapping of Product-Specific Detection Strategies

The preceding sections demonstrate that quality assessment requirements vary substantially across dairy product categories due to differences in composition, processing mechanisms, and dominant failure modes. These differences directly determine the suitability of sensing technologies and AI modeling strategies.

To facilitate systematic comparison, a comprehensive summary of product-specific quality risks and corresponding AI-enabled detection strategies should be presented in tabular form (Table 1). The comparison across dairy product categories indicates that the optimal sensing–AI strategy is determined primarily by the dominant quality attributes and failure modes of each product rather than by the nominal superiority of a particular algorithm. Raw milk is particularly suited to spectroscopy-based compositional and adulteration assessment; milk powder requires complementary chemical, spatial, and volatile information. Cheese benefits from combining spectroscopic or microbial screening with image-based assessment of ripening and surface defects. Yogurt requires greater emphasis on dynamic freshness, fermentation, and sensory-related attributes. Across all product categories, the major limitation is not the lack of high-performing algorithms but the limited evidence for transferability across products, batches, instruments, processing conditions, and industrial environments. This suggests that future research should move from isolated task-specific models toward validated sensing–AI systems that integrate multiple quality attributes and remain reliable under realistic operating conditions.

## 4. Explainable AI as the Trust Anchor in Dairy Quality Assessment

Rapid advances in artificial intelligence (AI) have substantially improved the efficiency and accuracy of dairy quality assessment. However, with the increasing complexity of AI models, the “black-box” nature of intelligent detection systems has become more prominent. Although these models can achieve high predictive performance, their internal decision-making logic is often difficult to interpret, making the detection results challenging for researchers, production operators, and regulatory agencies to fully understand and verify. Consequently, the development of intelligent detection systems that combine both high predictive capability and transparent reasoning has emerged as an important research direction in dairy AI. Explainable Artificial Intelligence (XAI) has therefore attracted increasing attention as a promising framework for enhancing the transparency and trustworthiness of AI-assisted dairy quality assessment. By revealing the internal reasoning mechanisms of AI systems, XAI enables detection results to evolve from merely “usable” toward “trustworthy and interpretable”.

Although XAI has been extensively explored in fields such as medical imaging, financial risk assessment, and autonomous driving, its application in dairy quality assessment remains relatively limited. According to the target of explanation, as shown in Table 2, XAI strategies in dairy detection can generally be categorized into three dimensions: input-level explanation, decision-level explanation, and output-level explanation. Input-level explanation focuses on identifying which original features contribute most significantly to model predictions, thereby revealing the information basis underlying model judgments. Decision-level explanation aims to clarify the internal reasoning paths and feature interaction logic that transform inputs into outputs. Output-level explanation emphasizes the confidence and uncertainty associated with prediction results, providing reliability information for practical decision-making. These three dimensions correspond to different explanatory objectives and methodological requirements in dairy quality assessment applications.

### 4.1. Input-Level Explanation

Input-level explanation focuses on the original feature information upon which model predictions rely. In spectroscopic detection tasks, this type of explanation is commonly reflected through importance analysis of characteristic wavelengths or spectral bands. By identifying the most influential variables, XAI methods can help researchers establish associations between AI decisions and underlying physicochemical properties, thereby improving both model transparency and scientific interpretability. Bao et al. [57] developed a U-Mamba framework integrating U-Net and Mamba modules for camel milk adulteration detection via near-infrared spectroscopy. SHAP analysis identified chemically meaningful spectral bands associated with water, protein, and fat, achieving 98.60% classification accuracy after hybrid generative augmentation. This establishes transparent associations between model decisions and underlying physicochemical properties, enhancing input-level interpretability.

### 4.2. Decision-Level Explanation

Decision-level explanation focuses on the internal reasoning paths within models, specifically how final judgments are formed from input features. Dairy assessment tasks usually involve complex nonlinear mapping, and multiple variables may be simultaneously relied upon by models due to multi-component coupling effects. Hu et al. [58] built an interpretable XGBoost model to discriminate yak milk feeding patterns using routine compositional parameters. SHAP, PDP, and ICE analyses revealed fat and lactose as the most discriminative features, uncovering meaningful interactions among fat, lactose, and freezing point with 92% accuracy and 0.94 AUC. This clarifies internal reasoning paths behind classification decisions, supporting decision-level explanation.

### 4.3. Output-Level Explanation

Output-level explanation focuses on the expression of confidence in model prediction results. In dairy detection, providing only classification results or numerical predictions is often insufficient to support practical decision-making. Output forms that include confidence levels, risk grades, or uncertainty intervals are more practically valuable. In adulteration detection tasks, in addition to judging whether samples are abnormal, the degree of prediction reliability should also be indicated by the model. Goyal et al. [59] developed an AI-enabled IoT multi-sensor system for real-time milk adulteration detection using pH, conductivity, temperature, gas, and VOC sensors. A voting ensemble classifier achieved 96% accuracy in detecting five common adulterants. SHAP analysis elucidated the ensemble decision-making process, enabling operators to understand how multi-sensor inputs are integrated into final judgments and enhancing trust in real-time outputs.

## 5. Challenges and Outlook

The integration of artificial intelligence and sensing technology has promoted the development of dairy quality detection toward automation and intelligence. Progress has been made by existing research in detection accuracy and algorithm innovation, but problems remain in data resources, model generalization capability, interpretability evaluation, and industrial adaptability. Future development needs to shift from algorithm-driven approaches toward comprehensive innovation supported by data and guaranteed by trustworthiness.

### 5.1. Data Challenges in AI-Driven Dairy Detection

Data serve as the foundation for artificial intelligence model construction, but dairy quality detection data are characterized by high acquisition difficulty, low standardization, and significant dynamic variation. The scarcity of high-quality annotated data is the primary problem. In compositional analysis tasks, labels must be obtained through laboratory chemical analysis. In microbial detection tasks, annotation involves lengthy cultivation and professional identification. For image-based defect detection, fine annotation by experienced domain experts is required. Some detection tasks inherently exhibit small-sample characteristics. For example, rare contamination events, special adulteration behaviors, and novel pathogen risk samples are difficult to obtain in large quantities in reality. This causes deep learning models to easily overfit and limits their ability to learn complex abnormal patterns. Few-shot learning, semi-supervised learning, self-supervised pre-training, and synthetic data augmentation are important technical pathways to alleviate this problem.

The lack of standardized public benchmark datasets is another structural obstacle. Laboratory-built datasets are generally relied upon by current research. Different studies vary significantly in sampling conditions, detection equipment, preprocessing procedures, and evaluation indicators. Consequently, fair comparison among different methods is difficult, unified validation benchmarks for model performance are lacking, and research results are difficult to reproduce. In spectroscopic and hyperspectral detection, differences in instrument brands, resolution, and preprocessing parameter settings may all affect model results. In the future, open dairy detection databases shared across institutions need to be constructed, and unified standards for data acquisition, preprocessing, and evaluation need to be established. These include public benchmark sets for raw milk quality, standard datasets for milk powder adulteration detection, and open databases for dairy microbial risk.

### 5.2. Challenges and Opportunities for XAI in Dairy Detection

The application of XAI in dairy quality assessment faces challenges that mirror those identified in broader XAI research. A primary issue is the trade-off between model performance and interpretability. Complex deep learning models achieve higher accuracy in tasks such as microbial colony classification and spectral regression, yet their hierarchical features resist direct mapping to physical or chemical attributes that dairy scientists recognize. Conversely, interpretable linear models or shallow decision trees struggle to capture the nonlinear relationships inherent in spectral data and complex food matrices [60]. Consequently, simplifying a model for better interpretability often reduces its predictive accuracy, while maintaining high performance in safety-critical applications demands transparent decision-making processes.

Current studies lack standardized metrics to assess whether an explanation is faithful to the model, stable across perturbations, and comprehensible to end users. Mersha et al. [60] emphasize that XAI systems require evaluation mechanisms measuring fidelity, consistency, robustness, and sufficiency. Brankovic et al. (2025) [61] further highlight that without domain-informed validation, explanations may appear actionable without being causally valid, or domain-relevant features may introduce fairness concerns. Their proposed CLIX-M checklist, which assesses domain relevance, reasonableness, and actionability, offers a framework that could be adapted for dairy quality control. For dairy applications, this means explanations must be checked against dairy chemistry principles to ensure mechanistic plausibility, rather than accepting mathematical importance as physical relevance.

A further limitation involves the potential for explanations to mislead rather than inform. Herrera [62] notes that post-hoc explanations can increase overreliance, particularly when users lack domain expertise to critically evaluate AI outputs. In dairy quality control, this risk is acute when operators accept AI recommendations without understanding underlying spectral or image features. Explanations must therefore be designed with the specific audience in mind, aligning with the expertise level of laboratory technicians or quality managers who make the final decisions.

### 5.3. Emerging Research Directions

Generative AI and foundation models represent a potential next stage in the development of AI-driven dairy quality assessment. Generative models, including GANs and transformer-based generative architectures, can be used to augment limited spectral or imaging datasets, simulate underrepresented quality conditions, and potentially support data-efficient model development. The emerging use of transformer-based GANs for NIR spectral generation and GAN-based spectral augmentation in microbial detection illustrates the feasibility of this direction. However, generated data must preserve the underlying physicochemical relationships of real dairy samples; otherwise, synthetic-data augmentation may introduce unrealistic patterns or amplify existing biases.

Foundation models may further shift dairy quality assessment from isolated task-specific models toward unified multimodal systems. Pre-training on large-scale datasets containing spectroscopy, hyperspectral images, conventional images, electronic-sensor signals, and physicochemical measurements could enable models to learn transferable representations across dairy products and quality-assessment tasks. Such models could subsequently be adapted to specific applications such as adulteration detection, freshness prediction, microbial-risk screening, or process monitoring with relatively limited labeled data. Nevertheless, dairy-specific foundation models remain constrained by the lack of large-scale standardized datasets, substantial computational requirements, domain-specific adaptation, and the need for reliable evaluation. Future studies should therefore investigate how generative AI and foundation models can be combined with physical constraints, domain knowledge, uncertainty quantification, and explainable AI to ensure that model outputs remain scientifically meaningful and operationally trustworthy.

A critical accompanying development will be the advancement of multimodal explainability techniques. As dairy assessment increasingly relies on heterogeneous data sources, future XAI methods must evolve beyond single-modality attribution to provide coherent, cross-modal explanations. This involves developing integrated attribution frameworks that jointly allocate importance across spectral wavelengths and image regions, offering unified mechanistic interpretations rather than isolated modality-specific analyses.

Finally, cross-instrument transferability represents a key frontier. Future models should be designed to generalize across different spectroscopic and imaging devices through domain adaptation and instrument-agnostic feature learning. Establishing standardized protocols for cross-laboratory model deployment will enable robust quality control frameworks that maintain performance regardless of equipment variations, advancing reproducible and scalable AI-driven dairy inspection.

## 6. Conclusions

This review provides a comprehensive analysis of the convergence between artificial intelligence and advanced sensing technologies in dairy quality assessment. By integrating data-driven AI algorithms into spectroscopic, bionic, and visual perception systems, detection methodologies have evolved from traditional destructive laboratory analysis toward automated, non-destructive, and intelligent frameworks. The incorporation of machine learning and deep learning has substantially enhanced detection accuracy, operational efficiency, and generalization capacity across complex dairy matrices, driving the transition from offline sampling to real-time, online quality monitoring. Concurrently, the emergence of explainable AI has addressed the critical transparency gap in black-box models, transforming detection outputs from merely “usable” to “trustworthy and interpretable” through feature attribution, reasoning clarification, and uncertainty quantification.

Despite these transformative advances, current AI-driven dairy detection systems still face fundamental challenges. The scarcity of high-quality annotated data, absence of standardized public benchmarks, and inherent trade-off between model complexity and interpretability continue to constrain industrial deployment. Cross-instrument variability and domain shift further limit model transferability across different production environments. Future efforts should focus on constructing standardized multimodal dairy datasets, developing lightweight pre-trained models for cross-instrument deployment, and establishing domain-grounded XAI evaluation frameworks. Advancing these directions will facilitate the transition from laboratory prototypes to scalable industrial applications, ultimately realizing trustworthy and intelligent dairy quality assurance.

## Figures and Tables

**Table 1:** Product-specific quality risks and AI-based detection strategies.

Product	Major Quality Risks	Technology	Representative Models
Raw milk	SCC, TBC, adulteration	NIR, HSI	SVM, CNN
Milk powder	Moisture, melamine, adulteration	NIR, HSI, E-nose	RF, CNN, Meta-learning
Cheese	Mold, ripeness, texture defects	NIR, Vision	RF, CNN
Yogurt	Freshness, fermentation, flavor	E-nose, Vision	LR, SVM, ResNet

**Table 2:** XAI methods and applications in dairy quality assessment.

XAI Level	Dairy Task	AI Model	XAI Method	Key Findings	Study
Input-level	Camel milk adulteration detection	U-Mamba	SHAP	Identified spectral regions associated with water, fat, and protein, improving physicochemical interpretability	[57]
Decision-level	Yak milk feeding pattern discrimination	XGBoost	SHAP, PDP, ICE	Revealed interactions among fat, lactose, and freezing point influencing classification decisions	[58]
Output-level	Real-time milk adulteration detection	Voting Ensemble	SHAP	Explained sensor contributions and improved confidence in prediction outputs	[59]

## Data Availability

No new data were created or analyzed in this study. Data sharing is not applicable to this article.
